# Early low complication rate of ceramic-on-ceramic total hip arthroplasty by direct anterior approach

**DOI:** 10.1051/sicotj/2020027

**Published:** 2020-08-04

**Authors:** Paul Henri Bauwens, Camdon Fary, Elvire Servien, Sébastien Lustig, Cécile Batailler

**Affiliations:** 1 Department of Orthopaedic Surgery, Lyon North University Hospital Lyon France; 2 Department of Orthopaedic Surgery, Western Health Melbourne Australia; 3 Australian Institute for Musculoskeletal Science (AIMSS), The University of Melbourne and Western Health St. Albans VIC Australia

**Keywords:** Total hip arthroplasty, Acetabular cup, Ceramic-on-ceramic, Complications, Direct anterior approach

## Abstract

*Introduction*: Ceramic-on-ceramic couplings are an alternative bearing surface to reduce the problems related to polyethylene wear and debris. However, ceramic articulations have their own risk of unique complications: fracture, squeaking, or dislocation. Few studies have assessed the outcomes of ceramic-on-ceramic total hip arthroplasties (THA) by direct anterior approach (DAA). The aim was to evaluate the early complications and revision rate of ceramic-on-ceramic THA by DAA. *Material*: A retrospective single-center study of 116 consecutive THAs was performed by DAA (106 patients) with ceramic-on-ceramic bearing from January 2015 to February 2018 with a minimum 24 months of follow-up. No patients were lost to follow-up. The mean age was of 55.3 years ± 11.3. The same cementless acetabular shell with a Biolox Delta ceramic insert and head were used. The complication and revision rates were collected at the last follow-up. The positioning of the acetabular implant was assessed on standard radiographs. Postoperative clinical outcomes were assessed by the Harris Hip Score. *Results*: At a mean follow-up of 31.9 months ± 5.5, no THA was revised. Five patients had late complications: 3 squeaking (2.6%) and 2 psoas impingements (1.7%) and were managed conservatively. All patients had satisfactory bony ingrowth of acetabular component, with no radiolucent lines and no osteolysis. Eight patients (6.9%) had an anterior overhang of the cup. The mean overhang for these patients was 4.1 mm. 111 hips (96%) were perceived as forgotten or having no limitations. *Conclusion*: This ceramic-on-ceramic coupling and shell by DAA produced excellent clinical outcomes and implant survival rate at a minimum two-year follow-up study. No serious complication was observed during the follow-up.

## Introduction

The number of total hip arthroplasty (THA) has risen sharply for several years (10% more in 4 years) [1]. This increase is considered to be related to both an aging population and an extension of THA indications, particularly in young and active patients. Both of these groups can have very different demands on their prosthesis such as longevity and avoiding dislocation or both. Ceramic-on-ceramic couplings are attractive alternative bearing surfaces. They have been reported to reduce problems related to debris from polyethylene wear following THA [2–5]. However, ceramic articulations have their own unique complications: fracture of the insert or the head [6, 7], squeaking [8, 9], and hip dislocation.

The direct anterior approach (DAA) had a low dislocation risk from posterior capsule and muscle preservation [10]. However, some papers report increased malpositioning with DAA techniques [11, 12]. Cup malpositioning is a cause of ceramic fracture and/or squeaking during activities of daily living. It is crucial to assess the early results of an implant particularly when implanting through a new technique that may increase the risk of cup malposition. Only one study has reported outcomes after ceramic-on-ceramic THA by DAA [10].

The purpose of this study is to assess the complications and the revision rate of a consecutive series of primary ceramic-on-ceramic THA couplings by direct anterior approach with a two-year minimum follow-up. Clinical outcomes and radiographic results were also assessed. Our hypothesis was that a ceramic-on-ceramic bearing in a cementless shell would produce excellent early clinical outcomes and implant survival rate.

## Material and method

### Patients

Between January 2015 and February 2018, 873 consecutive THAs were performed in our department. In this cohort, 640 were performed by a direct anterior approach (73%) and 233 were performed by a posterior approach (27%). This retrospective and single-center study included 116 consecutive THAs performed by DAA (106 patients) with the same acetabular cup and ceramic-on-ceramic coupling. All patients had a minimum of 24 months follow-up. Nine patients had bilateral THA at the same time and one with a period of 6 months between the two sides.

The mean age was of 55.3 years ± 11.3 [23.3; 76.8]. The mean body mass index (BMI) was 25.8 kg/m^2^ ± 4.4 [17.7; 39.3]. There were no patients lost to follow-up and no excluded patients. Demographic data and indications for surgery are reported in Table 1.

**Table 1:** Demographic characteristics and preoperative scores for THA.

	No THA (%)	Mean ± SD [min; max]
No. of hips (no. patients)	116 (106 patients)	
Age (yo)		55.3 ± 11.3 [23.3; 76.8]
Age category (%)		
< 40 yo	13 (11.2%)	
40–65 yo	81 (69.8%)	
> 65 yo	22 (19.0%)	
Gender (Men)	55 (47.4%)	
BMI (kg/m^2^)		25.8 ± 4.4 [17.7; 39.3]
Side (right)	61 (52.6%)	
Previous surgery (hips)	4 (4.6%)	
Preoperative diagnosis (hips)		
Osteoarthritis	105 (90.5%)	
Hip dysplasia	5 (4.3%)	
Femoral neck fracture	4 (3.4%)	
Sepsis	2 (1.7%)	
HSS		50.1 ± 10.7 [14; 71]

SD: Standard Deviation; yo: years old; HSS: Harris Hip Score.

### Implants and surgical technique

All the 116 THAs included were performed by a single surgeon using the DAA approach of Hueter Gaine [13, 14] with a standard orthopedic table, as described by Lustig et al. [15]. A routine accelerated post-op recovery protocol was performed (mobilization on the day of surgery, optimized pain treatment, fast discharge at 1 or 2 day postoperatively after therapeutic education …). No drain was used. Intraoperative fluoroscopy was used to confirm appropriate positioning of implants.

**Figure 1:**
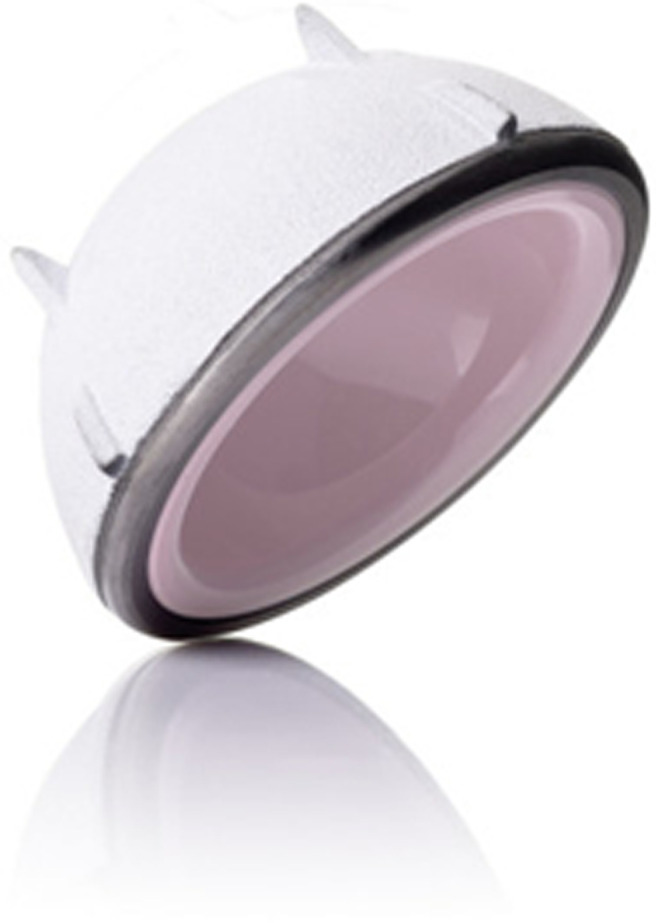
Ceramic-on-ceramic acetabular cup (CARGOS™ HAP Press Fit cup) with equatorial fins and tropical spikes.

The implants were cementless. The femoral stem was forged titanium alloy, fully coated with nonporous hydroxyapatite (Targos stem with collar, Lépine, France). The titanium alloy CARGOS™ HAP Press Fit cup is a cementless acetabular cup (Lépine, France) with a bioactive titanium-hydroxyapatite coating is applied using a vacuum plasma spray technique (Fig. 1). A Biolox Delta ceramic insert made of high purity alumina is secured by a conical assembly. The cup has equatorial fins and tropical spikes to increase stability and prevent rotation and tilting before secondary osseous integration occurs.

A 32-mm diameter ceramic head was used in 35 hips (30.2%) and a 36-mm diameter ceramic head was used in 81 hips (69.8%). A 36-mm diameter head was used with shell size greater or equal to 50 mm.

### Data collection

The demographic data (age, gender, BMI, etiologies) and a pre-operative Harris Hip Score (HHS) were collected. Postoperative clinical assessment was evaluated using the Harris Hip Score (HHS) by an independent observer. The results were classified as: poor (< 70), fair (70–79), good (80–89), and excellent (90–100). The satisfaction following THA was also recorded (very satisfied, satisfied, disappointed, dissatisfied) by an independent observer.

On the postoperative pelvic AP views, the measurements of implant positioning were performed by an independent observer. Cup abduction was the angle between the cup axis and the parallel between the inter-teardrop line. The anteversion was calculated using Widmer’s method [16]. The cup positioning was considered satisfactory if within Lewinnek’s safe zone [17]: abduction between 30° and 50° and anteversion between 5° and 25°.

### Ethical approval

All procedures performed in studies involving human participants were in accordance with the ethical standards of the institutional and/or national research committees and with the 1964 Helsinki declaration and its later amendments or comparable ethical standards. For this type of study formal consent is not required. The Advisory Committee on Research Information Processing in the Field of Health (CCTIRS) approved this study on June 4, 2015 under number 15-430.

### Statistical analysis

For the statistical analysis, the XLs-stat software (2015; Addinsoft) was used. Both univariate and multivariate analyses were performed to assess predictive factors for implant positioning and complications. The continuous variables were averaged and reported with standard deviation and interval. The potential predictive factors assessed were BMI, age, gender.

The survival rates were calculated using the Kaplan–Meier method with a 95% confidence interval. A *p*-value < 0.05 was considered statistically significant in each analysis.

## Results

None were lost to follow-up or died at a mean follow-up of 31.9 months ± 5.5 [24; 42.5].

### Complications

Intra and post-operative complications are reported in Table 2. None of the 106 patients had a hip revision. Five patients had minor complications: 3 squeaking (2.6%) and 2 psoas impingements (1.7%). Physiotherapy and rehabilitation managed these complications conservatively. There was no dislocation or ceramic fracture.

**Table 2:** Intra and post-operative complications of THA, and post-operative functional outcomes at the last follow-up.

Complications	*N* = 116 THA
Surgical revision	0
Major complications	0
Dislocation	0
Femoral fracture	0
Acetabular fracture	0
Infection	0
Minor complications	9 (7.8%)
Psoas impingement	2 (1.7%)
Squeaking	3 (2.6%)
Greater trochanter fractures	3 (2.6%)
Femoral wrong way	1 (0.9%)
Functional outcomes	
HSS	
(Mean ± SD) [min; max]	92.4 ± 6 [70; 100]
Repartition of HSS	
Excellent (> 90)	99 (85.3%)
Good (80–89)	12 (10.3%)
Fair (70–79)	5 (4.3%)
Poor (< 70)	0

SD: Standard Deviation; HSS: Harris Hip Score.

The Kaplan–Meier survivorship at 31.9 months was 100%.

### Functional outcomes assessment

The functional outcome scores are reported in Table 2.

Functional outcomes study with Harris Hip Score showed that patients’ satisfaction was excellent in 99 hips. 111 hips (96%) were perceived as forgotten or having no limitations.

### Radiographic assessment

**Figure 2:**
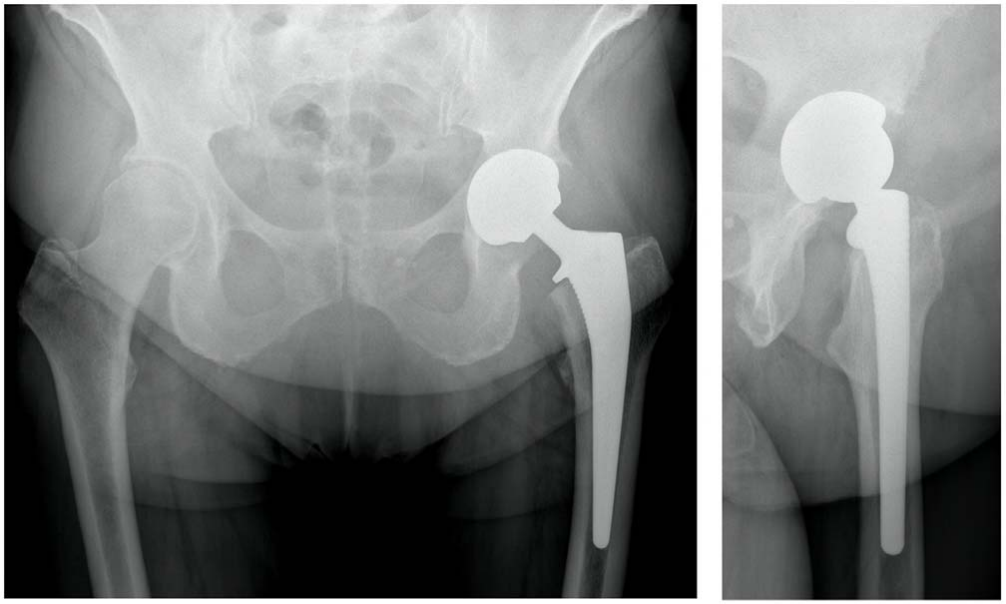
Antero-posterior pelvic radiograph and profile left hip radiograph of a patient with psoas pain, despite a satisfying cup positioning without cup overhang.

Implant positioning is summarized in Table 3. Eight patients (6.9%) had an anterior overhang of the cup. The mean overhang for these patients was 4.1 mm. No overhang was greater than 10 mm. The patients with an anterior overhang had no psoas pain and no shell revision was necessary for this overhang. The two patients with psoas impingement had a satisfying cup positioning without cup overhang (Fig. 2).

**Table 3:** Acetabular implant positioning at the last follow-up.

Implant positioning	*N* = 116 THA
Cup inclination (mean ± SD) [min; max]	44.9 ± 5 [30.6; 60]
Zone < 30°	0
Zone > 50°	11 (9.5%)
Cup anteversion (mean ± SD) [min; max]	16.4 ± 3.7 [9; 30]
Anterior overhang of the cup	8 (6.9%)
Mean anterior overhang (mm) (mean ± SD) [min; max]	4.1 ± 2.1 [1.1; 7.1]

All patients showed satisfactory bony ingrowth of the acetabular component, with no radiolucent lines and no osteolysis. All acetabular cups were seated to the level that the acetabulum was reamed.

There was no correlation between BMI and the cup positioning.

## Discussion

We have found that a ceramic-on-ceramic bearing in a cementless shell produced excellent early clinical outcomes and implant survival rate at a minimum follow-up of 2 years. There were no major complications or surgical revision required.

One of the main concerns of ceramic-on-ceramic articulation is the risk of fracture of the ceramic liner and/or head. We have not encountered ceramic fractures or otherwise with this implant design by DAA. The causes of ceramic fracture are attributed to imperfection in production of the ceramic, malpositioning of the ceramic insert into either the acetabular cup or malpositioning of the acetabular cup itself. Due to the significant improvements of ceramic production and quality, there have been significant decreases in the rate of ceramic head fractures [5, 18]. New-generation ceramic liners do not fracture at an impact force of 12 kN, a force greater than most estimates of the physiological forces to which the hip is subjected during falls or stumbling [19]. The usual activities of normal life are within 12 kN and fracture without trauma is unlikely [20]. In literature, some studies reported ceramic fracture after high energy trauma [21], others reported that it could occur without high energy trauma [7, 22, 23]. A malpositioning of the ceramic insert within the metal back of the cup or malpositioning of the cup will increase the risk of ceramic fracture with low energy trauma [24]. Acetabular shell malpositioning can be due to insufficient exposure of the acetabulum during surgery. Technical problems involving inadequate positioning of the ceramic liner in the acetabular shell or a difficult reduction during the surgery from high soft-tissue tension can make a chip fracture of the ceramic insert also [25–27]. Ceramic fragments from the insert may produce excessive wear, and the localized impact of an insert may increase the risk of its fracture. A cup that is too vertical increases point loading on the insert and femoral head, then increases the risk fracture [26]. These are all possible causes for early ceramic failure and fractures [28].

The difficulties of exposure by the DAA and the risk of complication are described in literature [29–31]. The most commonly reported complication of DAA is femoral fracture or malposition of the femoral implants [32–34]. In literature, some cases of acetabular complications, during the surgery or at long term, have been reported with DAA, the acetabular exposure being relatively convenient by DAA. Only one study has reported outcomes of ceramic-on-ceramic THA by DAA [10]. They reported no significant difference in rate of ceramic-on-ceramic coupling complications between posterior approach and DAA without further details of the actual complications that did occur. Our study demonstrates that the DAA with our approach has few complications of a ceramic-on-ceramic bearing. We believe that intraoperative fluoroscopy during surgery is important to prevent acetabular malpositioning.

Repetitive impaction of a ceramic femoral head on the rim of a ceramic liner following recurrent dislocation or subluxation is another possible cause of a chip fracture [35]. An advantage of DAA is the low risk of hip dislocation [10, 36–38]. These results confirm that the dislocation risk is very low, without dislocation described in this series.

The treatment of a ceramic implant fracture remains controversial [18, 35, 39–42]. Several studies have recommended the use of ceramic-on-ceramic bearings when revising a ceramic fracture as ceramic particles could produce rapid third-body wear in soft polyethylene. However, it is important to replace a ceramic head onto a used or damaged Morse taper due to a mismatch between the metal taper and the bore of the head, stress concentrations, and important risk of head refracture [18, 40, 41]. If a metal sleeve to cover the trunnion and avoid a mismatch is not available, the next option is to change the femoral stem, but removal of well-fixed stem can be a complex surgical procedure and not without a significant complication or morbidity of its own that needs to be taken into consideration. Other studies have reported revision with a cobalt–chromium head and a polyethylene insert with good outcomes [21, 42]. At our institution in THA revisions our preference is a dual mobility cup to limit the instability risk [43].

We found a squeaking rate of 1.7% (3/116) in our study. Squeaking THA ceramic bearings have been reported in 0.3% to 23% [9, 44, 45]. Chevillotte et al. [8] described that squeaking in ceramic-on-ceramic joint may be due to the disruption of fluid lubrication. In another study [46], they reported that a mismatch between zirconium head and an alumina liner can be squeaking. Other authors have not found an obvious risk factor for squeaking [28]. Blakeney et al. reported that squeaking was significantly associated with younger age, larger head diameter, higher UCLA score, and SF-12 PCS score [45]. The majority of squeaking THA are asymptomatic and not require revision [47]. Occasionally squeaking can be due to undisplaced ceramic fracture and can change in frequency and intensity with time [48]. Squeaking did not affect satisfaction of patient in our study, with all of the squeaking hips remaining satisfied by the surgery and there was no common risk factor found.

Primary acetabular stability in rotation and tilting of our shell is improved with equatorial fins and tropical spikes enabling a good pressfit, and by the fact that the shell is a larger circumference than the reamer; but may be difficult to position for a surgeon with less experience. We had 8 (6.7%) anterior overhangs of which 5 occurred within the first year. In DAA literature, the mean anteversion and acetabular abduction ranged from 19° to 27° and from 39° to 47°, respectively, depending on the author targets [10, 11, 49, 50]. With our DAA technique appropriate abduction and anteversion of the acetabular reamer is along the axis of the incision which enables the use of a standard straight instrumentation [12]. Our THA functional outcomes are similar to other studies [10, 11, 49, 50].

This study has limitations. A limitation is that the assessment of implant position was on radiograph and not CT scan. Consequently, some measurements may be less accurate, particularly the cup anteversion. Nevertheless, a CT scan is not the standard postoperative test after THA. This would have exposed patients to radiation and do not reflect common practice. The quality of the radiographs could be the second limitation, but they are performed in the same department by X-ray technicians specialized in the lower limb. This study is also retrospective. Nevertheless, the main aims were to assess implant position and complications, which were not influenced by retrospective analysis. Follow-up time was short, and results could not be extrapolated to longer term. But to our knowledge, this study is the first to specifically assess and report outcomes of delta ceramic-on-ceramic by DAA. Assessment of early results and complications of prosthesis is important before widespread use is adopted, and it may have implications for development of prosthesis design and further innovation.

## Conclusion

This ceramic-on-ceramic bearing performed by direct anterior approach produced excellent clinical results and implant survival rates at a minimum of two years of follow-up. No major complication was observed during the follow-up, particularly no ceramic fracture or dislocation. Ongoing assessment of complications, further innovation, and revision rates particularly in the long term will be important.

## Conflict of Interest

The authors received no specific funding for this work. Each author certifies that he or she has no commercial associations (e.g. consultancies, stock ownership, equity interest, patent/licensing arrangements, etc.) that might pose a conflict of interest in connection with the submitted article.

## Funding

There is no funding source.

## Ethical approval

All procedures performed in studies involving human participants were in accordance with the ethical standards of the institutional and/or national research committee and with the 1964 Helsinki declaration and its later amendments or comparable ethical standards. The Advisory Committee on Research Information Processing in the Field of Health (CCTIRS) approved this study on June 4, 2015 under number 15-430. For this type of study formal consent is not required.
